# ‘Relieved Working’ study: systematic development and design of an intervention to decrease occupational quartz exposure at construction worksites

**DOI:** 10.1186/1471-2458-14-760

**Published:** 2014-07-28

**Authors:** Karen M Oude Hengel, Erik van Deurssen, Tim Meijster, Erik Tielemans, Dick Heederik, Anjoeka Pronk

**Affiliations:** Netherlands Organisation for Applied Scientific Research TNO, P.O. Box 718, 2130 AS Hoofddorp, The Netherlands; Utrecht University, IRAS (Institute for Risk Assessment Sciences), Utrecht, The Netherlands

**Keywords:** Occupational quartz exposure, Construction industry, Intervention mapping

## Abstract

**Background:**

Occupational quartz exposure continues to be a serious hazard in the construction industry. Until now, evidence-based interventions aimed at reducing quartz exposure are scarce. The aim of this study was to systematically develop an intervention and to describe the study to evaluate its effectiveness.

**Methods/Design:**

The intervention was developed according to the principles of the Intervention Mapping protocol, meaning that evidence from the literature was combined with information collected from stakeholders (e.g., construction workers, managers and researchers). The intervention aimed to integrate technical, behavioural and organizational factors. The intervention consists of two plenary meetings for all employers within the company, and individual visits at construction worksites, including specific intervention materials. Additionally, a demonstration session regarding control measures was organized for all managers. The effectiveness of the intervention will be evaluated in a cluster randomized controlled trial among eight construction companies, with measurements at baseline and follow-up. Outcome measures are personal respirable dust and quartz exposure by means of exposure assessment, and behavioural and organizational determinants which will be assessed by means of questionnaires. Additionally, a process evaluation will shed light on whether the intervention (does not) works, and, if so, the reasons for this.

**Discussion:**

Applying Intervention Mapping in the development of an intervention to reduce occupational quartz exposure was useful, as different stakeholders provided input for the intervention as well as the implementation strategy. Therefore, the feasibility of the intervention has been enhanced, as it appeals to construction workers and managers and will not unduly interfere with the ongoing construction work.

**Trial registration number:**

NTR4586 (May 7th 2014).

## Background

Occupational exposure to respirable dust containing quartz continues to be a serious hazard in the construction industry, as it experiences high quartz exposure levels during specific activities [1–6]. This is illustrated by previous studies which showed exposure levels well above the occupational exposure limits (OEL) for quartz among several jobs and tasks in the construction industry [1, 3, 5–7]. These high exposure levels could be explained by the fact that construction workers regularly work with building materials such as concrete and lime-sandstone, of which quartz is a major constituent.

As quartz exposure seems inevitable in construction work, construction workers could face potential health risks, mainly lung diseases such as silicosis and Chronic Obstructive Pulmonary Disease(COPD) [4, 8–10], due to chronic occupational quartz exposure at an elevated level [11, 12]. Quartz has been classified as a carcinogen, and silica exposure also contributes to lung cancer morbidity and mortality [13, 14]. Considering these potential health risks for construction workers, the need for evidence-based approaches to reduce occupational exposure has been recognized within the political system in the Netherlands [15]. To date, technical control measures with Local Exhaust Ventilation (LEV), or wet suppression and personal respiratory protective equipment are most common to prevent occupational quartz exposure among construction workers [16]. Although experimental studies showed a reduction of quartz exposure when using these technical control measures properly [16, 17], there are few studies on the effectiveness of technical control measures under real working conditions. It could be hypothesized that it is more challenging to use technical control measures properly in practice, as construction worksites are temporary, and mobile, and variable in regard to job tasks and workspace [18, 19]. Thus, to implement effective interventions to reduce occupational exposure at worksites, interventions should not only focus on the implementation of technical control measures, but also take into account workers’ behavior and the organizational work environment [20].

The current study aimed to systematically develop an intervention targeting the integration of technical, behavioural and organizational factors in order to reduce occupational quartz exposure in the Dutch construction industry, through an application of the Intervention Mapping protocol [21]. The Intervention Mapping protocol was applied to systematically incorporate empirical findings from the literature and input from all stakeholders (e.i., workers, managers and researchers) into an intervention tailored to the construction workers. This paper describes Phase I of the study, the systematic development of the intervention, and the evaluation study regarding the effectiveness of this program (Phase II).

## Methods

The present study is divided in two phases. In the first phase, an intervention was systematically developed based on the intervention mapping approach, which describes a process for developing theory- and evidence-based intervention programs [21]. The second phase of this study involves the description of the evaluation of the intervention.

## Phase I: Intervention development

**Figure 1:**
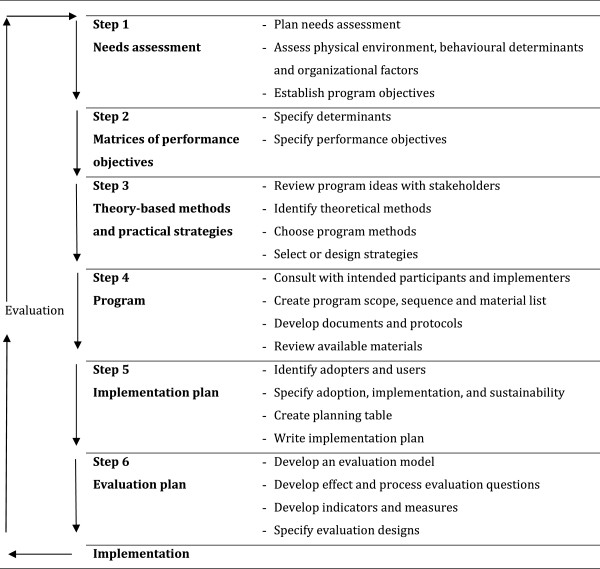
**Intervention mapping, source: Bartholomew et al.** [21] **.**

Intervention mapping facilitates a stepwise process to guide researchers through the development and planning of interventions by combining scientific knowledge and opinions from stakeholders (Figure 1). Intervention mapping involves a systematic process that prescribes six steps: (i) needs assessment; (ii) performance objectives and determinants; (iii) selection of methods and strategies: (iv) design of the intervention program; (v) development of a plan for implementation; and (vi) evaluation. The completion of these steps serves as a blueprint for the intervention in the current study.

### Step 1: Needs assessment

In step 1 of the intervention mapping approach, insight into the underlying factors causing elevated levels of occupational quartz exposure was gained. Ultimately, this insight resulted in the formulation of the program objective. This objective specifies what changes are needed to decrease the elevated quartz exposure level.

Insight into the underlying factors was achieved by means of (i) a questionnaire and an exposure assessment survey among construction workers; (ii) a questionnaire among managers; (iii) a literature review; and (iv) meetings with managers and sector organizations in the construction industry.

The main resource for step 1 was an assessment of the baseline characteristics of the study population. This assessment focused on the role of behavioral and organizational factors in relation to quartz exposure among construction workers [22]. The questionnaire distributed among construction workers was completed by 116 eligible participants, and produced insights in the behavioral factors. To explore the role of organizational factors, eight managers completed a questionnaire to obtain insight into the occupational safety and health policy of a company, using an adapted version from the Occupational Health and Safety Series (OHSAS) norm on hazardous circumstances [23]. Additionally, a literature search was conducted in Pubmed, using keywords relevant for the target population (e.g., “construction workers”, “construction industry”, “concrete driller” and “demolisher”) and outcome measures (e.g., “occupational exposure”, “quartz”, and “silica”). Furthermore, two focus groups were organized: one group with seven researchers and one focus group with three managers and three representatives of sector organizations. Three additional face-to-face interviews with managers of participating construction companies were also organized.

#### Factors related to occupational quartz exposure

Based on available resources, the factors that were associated with occupational quartz exposure were divided into work environmental factors, worker-related factors, and organizational factors.

Firstly, work environmental factors were divided into technical control measures and work activities. Several types of technical control measures to reduce quartz exposure such as local exhaust ventilation (LEV) or wet suppression are currently available for different job categories and job tasks in the construction industry [6, 16, 24, 25]. The availability of technical control measures varies among job categories. Concrete drillers, demolishers and tuck pointers have more access to technical control measures than other construction workers. Although experimental studies showed a significant exposure reduction by using these technical control measures, the use of technical control measures varied among the construction workers in the current study. The baseline study illustrated that ”maintaining a good health” and “inconvenience for eyes and airways” were mentioned by construction workers as reasons to work with these technical control measures [22]. Workers mentioned tools with poor ergonomic design, potentially resulting in a lower productivity, as constraints to work with technical control measures [22]. A previous study showed that using technical control measures with wet suppression resulted in wet materials or in a slippery working area [24]. Workers’ opinions regarding the use of technical control measures were recognized by managers and sector organizations. Managers also mentioned the difficulties in using technical control measures, considering the variety in working tasks, which asked for different technical control measures. Additional to the technical control measures, specific work activities are associated with higher occupational quartz exposure. The baseline study showed that working inside and working with cement slots was associated with a higher exposure to both respirable dust and quartz, whereas determinants like near-field worker-source distance and work orientation at shoulder level were associated only with higher respirable dust exposure [22]. Working in the same workspace with another worker and the method of handling dusty rubbish were mentioned during the focus groups as risk factors for elevated exposure. Lastly, meteorological conditions such as rain, and wind direction and speed, are also generally considered to be important determinants of inhalation exposure [26, 27].

Secondly, worker-related factors such as behavioral determinants influence whether construction workers use technical control measures. Previous studies showed that knowledge and theoretical competences about the proper use of technical control measures (e.g., connecting hoses and removing bags correctly, good sealing, maintenance and cleaning) are needed to maximize efficacy in controlling exposure [28–30]. Additionally, the baseline study showed that more peer pressure or presence of supervisors increased the use of technical control measures [22]. All participants in the focus groups mentioned that greater awareness and risk perception regarding the long-term health consequences of being exposed to dust and quartz potentially increases the use of technical control measures. They also emphasized improving the skills of construction workers to correctly implement these technical control measures.

Third, organizational factors include the responsibility of companies and sector organizations for reducing high level of occupational quartz exposure. First, the availability of technical control measures at worksites is the responsibility of companies. However, the majority of managers face difficulties in collecting the relevant information about technical control measures from the different resources such as labour inspection and sector organization. They therefore recommend providing a clear overview of the newest technical control measures and their functionality. The majority of employers also mentioned the increasingly shorter lead times for construction processes, and working with subcontractors led to tighter bids. This resulted in less time and investments to use technical control measures at construction worksites. Additionally, managers are responsible for organizing training sessions to educate their construction workers about quartz exposure and technical control measures. Education, procedures, and training sessions at the worksites are of particular importance for the current study population, as most of them work without any professional education. The baseline study showed that only 25% of the managers offered this kind of training/education and five of eight (63%) companies stated that compliance with work procedures and workplace instructions regarding dust-reducing practices was supervised by their management [22].

#### Program objective

Based on the needs assessment, the following program objective was formulated: establish an increase in the (proper) use of technical control measures in the construction industry by targeting both the worker’s behaviour and organizational factors in order to significantly reduce occupational respirable dust and quartz exposure.

### Step 2: Performance objectives and determinants

The performance objectives constitute the specific measurable objectives of the intervention program that are required from the target group (i.e., construction workers and managers) to achieve the program objective as described in the needs assessment. The results of the needs assessment showed that both the organization and workers’ behavior should be targeted adequately in order to increase the proper use of technical control measures at construction worksites. Three researchers of the project team (KOH, EVD and TM) discussed the results of the needs assessment and translated these into specific measurable performance objectives for both managers and construction workers to achieve during the intervention study. At the end, a list of performance objectives was constructed that fits the program objective of step 1. The final performance objectives were selected during an expert meeting with six researchers having broad experience in the field of occupational (quartz) exposure and/or implementation of interventions among blue collar workers (Table 1).

**Table 1:** **Performance objectives for individual and environmental changes related to reducing occupational quartz exposure**

What construction workers will do to reduce occupational quartz exposure	
1	Be aware of the risks and consequences of high occupational quartz exposure
2	Increase their knowledge on when they are exposed to occupational quartz exposure
3	Increase their knowledge about the causes and possible long-term risk of occupational quartz exposure
4	Identify solutions regarding the reduction of occupational quartz exposure at the worksites
5	Identify constraints for using technical control measures
6	Learn how to use technical control measures in a healthy and safe way
7	Discuss with colleagues their responsibility to reduce occupational quartz exposure
*What managers will do to change the work environment in order to reduce occupational quartz exposure*	
1	Have a positive attitude towards reducing occupational quartz exposure
2	Incorporate knowledge and solutions regarding reducing occupational quartz exposure in their policies
3	Provide technical control measures for construction workers to reduce occupational quartz exposure
4	Increase their knowledge about the causes and possible risks of occupational quartz exposure
5	Be responsible for the usage of the technical control measures in their company

Besides the performance objectives, changeable determinants of the performance objectives were selected during the expert meeting to facilitate a change in the behavior of construction workers and managers. As previously mentioned in the needs assessment, the following behavioral determinants at worker level were identified: knowledge, awareness, risk perception and skills. Knowledge and attitude were also defined as important behavioral determinants for managers. Additionally, social-cultural and economic determinants (e.g., investment in tools) were selected as organizational determinants. By defining the performance objectives and determinants, very specific intervention goals and materials could be developed in the next step of intervention mapping.

### Step 3: Selection of methods and strategies

After defining performance objectives and determinants, appropriate theoretical methods were selected to monitor behavioral and organizational changes and to translate these into practical strategies.

#### Theory-based intervention methods

For each determinant defined in step 2, appropriate theoretical methods were identified from literature and from the guidelines published by Bartholomew et al. (2006) [21]. A theoretical method describes the expected association between an intervention action and a change in identified individual and organizational determinants. Decisions regarding suitable theoretical methods were made, based on feedback from key contacts of the companies and sector organizations, as well as within the research group. Because the program outcomes and determinants are at individual and organizational level, theoretical methods were directed to both workers and managers. For example, as demonstrated in Table 2, scenario-based risk information was selected as a theoretical method to improve risk perception of both construction workers and managers in order to work with technical control measures.

**Table 2:** **Theory based methods, practical strategies and selected tools and materials for the intervention to reduce occupational quartz exposure**

Determinants	Theory based methods	Practical strategies	Selected training, tools and materials
**Behavioral determinants**			
Awareness	Passive learning	Providing (written) information	Personal letter to workers with information regarding the exposure levels of the baseline measurement
	Modelling	Role modelling	Poster for managers and workers
	Stimulating communication	Discussion	Interactive group sessions to exchange constraints/facilitators of using technical control measures
Knowledge	Passive learning	Providing (written) information	Tailored factsheets for workers
			Occupational physician will attend first meeting to explain health-risks
	Active Learning	Discussion	Interactive presentations including documentary and PIMEX during sessions at the worksite
		Demonstration	
			Interactive group sessions at the worksite
			Discussion with labour inspector regarding the context of using technical control measures
			Tailored sessions for managers to demonstrate technical control measures
	Images	Providing verbal information/images	Video on long-term effects of occupational exposure
Risk perception	Scenario-based risk information	Recognizing invisible risks	Video on long-term consequences of occupational exposure
			Occupational physician will attend first meeting to explain health-risks
		Recognizing behaviour	Pimex videos^1^
Skills	Feedback	Feedback	Group discussions at the worksite
	Guided practice	Guidance sheet	Tailored factsheets
			Tailored sessions for managers to use technical control measures
	Modelling	Demonstration	PIMEX videos^1^
**Organizational determinants**			
Social-cultural	Shifting focus	Workers telling their stories	Video on long-term consequences of occupational exposure
	Building skills for resistance	Discussion	Defining skills and reasons for resistance
	Reinforcement	Remembrance through incentives	Posters
	Social support	Discussion	Managers and workers discuss about constraints and facilitators to use technical control measures
Economic	Availability	Approval	Manager approves training during working time
			Managers are willing to invest in technical control measures

^1^PIMEX videos: Picture Mix Exposure, a video exposure monitoring method for exposure of hazardous materials to measure the impact of workplace practices and conditions at the same time.

#### Practical strategies

Theoretical methods were translated into practical strategies during two brainstorm sessions with the research team, and fine-tuned during the meeting with the key-contacts of the companies and sectors organizations. Specific tools and materials that fit the chosen determinants, theories and strategies were selected (Table 2). For instance, passive learning, modelling and stimulating communication were chosen as theoretical methods to increase the awareness of the consequences of occupational quartz exposure among construction workers and managers. Then, written information, role modelling and group discussion were selected as providing practical strategies that fit the population of construction workers and their managers. Specifically, visual program materials such as films and posters were developed for the study population in the current study, as it was mainly comprised of low-educated workers. Demonstrating and practicing with tools were chosen as the most suitable intervention session for managers to increase their knowledge.

### Step 4: Design of the intervention program

The first intervention program was subjected to commentary during an expert meeting with researchers, managers from larger companies, and representatives of sector organizations. Based on this meeting, a final version of the intervention program based on the tools and intervention materials from Table 2 was developed.

#### Program description

**Figure 2:**
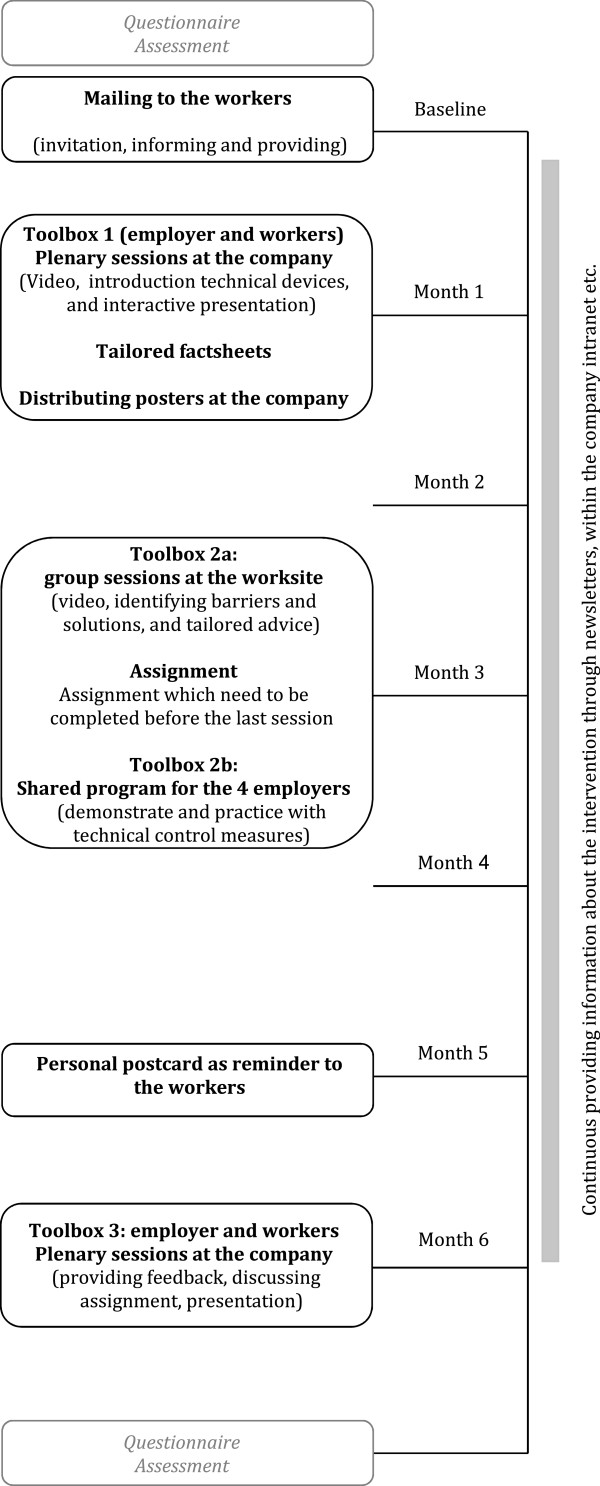
**Flow chart of intervention program.**

The intervention, so called ‘Relieved Working’ study, takes place during a six-month period and consists of plenary sessions and intervention materials (Figure 2).

The starting point of the six-month intervention is a personal letter to the construction workers in the intervention group, in which they are invited to the first plenary session and are notified for their exposure levels as obtained from the baseline survey. Employers receive a letter with company-specific average exposure levels, including an explanation regarding the intervention program and the role of managers.

#### Intervention program and materials

The first plenary session for all employees at the company (e.g., construction workers, managers and supervisors) was an interactive presentation by the principal researcher (EvD) and an occupational physician, consisting of the following elements: (i) showing a short documentary to increase risk perception and awareness of long-term health consequences; (ii) providing results of the baseline study, including both exposure and observed behavior; (iii) providing information regarding the health-risks; (iv) discussing situations with high quartz exposure; (v) teaching skills regarding the proper use of technical control measures by showing PIMEX videos (i.e., Picture Mix Exposure, a video exposure monitoring method for exposure of hazardous materials to measure the impact of workplace practices and conditions at the same time); (vi) discussing constraints and facilitators to work with technical control measures; and (vi) set tailored goals for the intervention period. At the end of this session, posters were distributed to companies and compact-sized factsheets are handed out to construction workers. For each job category, this tailored factsheet provides specific instructions on how to reduce quartz exposure at the worksite. These instructions are focused on (i) proper use of technical control measures; (ii) organizational factors such as asking the supervisor for technical control measures, and using protective equipment; and (iii) behavioral factors such as cleaning the workspace.

The second individual session is organized at the worksite and aims to teach workers how to use technical control measures in a proper and productive way. Therefore, the construction workers (i) define situations with high levels of exposure at their current worksite; (ii) learn how technical control measures should be used to reduce this exposure; (iii) discuss constraints for using technical control measures; and (iv) define solutions to tackle these constraints. At the end, an assignment is given to the construction workers in which they are asked to photograph two situations at the worksite (i.e., a good and a bad practice) which serve as input for the last plenary sessions.

Simultaneously with the worksite visits, a meeting is separately organized for the managers to gain more insight in the availability of technical control measures. This meeting consists of a demonstration of the newest available technical control measures, their innovations and their possibilities. As this session is held at the lab within the research institute, the managers have the possibility to practice with technical control measures and to discuss possible constraints related to functionality.

During the last plenary session at the company, managers and workers discuss, evaluate, and reconsider the goals and actions that are achieved. During an interactive presentation of the principal researcher, lessons learned and key-solutions to overcome the main constraints are discussed. The assignment with photos serves to open this discussion regarding the good and bad practices across different worksites. Additionally, a labour inspector joins this session to give practical information on their policy regarding quartz exposure.

### Step 5: Development of a plan for implementation

Researchers pay attention in step 5 to important preconditions that ensure optimal implementation and adoption of the ‘Relieved Working’ study. Firstly, as managers and supervisors play a key role in successful implementing an intervention at worksites [31], they are encouraged to actively participate in the intervention. Managers are also invited for a session with all participating companies to share ideas and learn about new innovations of technical control measures. Secondly, as time, place and costs are important factors to successfully adopt and implement the program [32, 33], the three sessions are organized within the existing so-called “Toolbox Education system” in the Dutch construction industry. The toolbox education system consists of at least 10 obligatory health and safety training sessions for workers, which have to be organized by managers in the construction industry each year in order to obtain an official health and safety certificate. Thirdly, because the intervention is implemented across different companies, a protocol including an outline, time schedule, and communication between all stakeholders was developed to standardize the intervention.

## Phase II: Evaluation of the intervention

### Step 6: Evaluation plan

#### Study design

The effectiveness of the intervention will be measured by performing a cluster randomized controlled trial (cluster RCT). Participants who have given informed consent are measured at baseline (T0), and will be measured after the intervention (T1). Workers at the worksite allocated to the intervention group receive the worksite intervention program during a six month period, while those allocated to the control group receive no intervention. The study protocol and measurements are not part of the judgement of the Central Committee of Research Involving Human Subjects, meaning no medical ethic approval is required for this study. The study has been executed according to the Dutch Data Protection Law.

#### Study population

The study population includes blue collar workers in the construction industry. These workers are recruited through eight companies specialized in either demolishing, concrete drilling, sawing and/or façade maintenance, which are all activities associated with relatively high silica exposure levels. Inclusion criteria for the construction workers are (i) available for the intervention period; (ii) sufficient competency in the Dutch language; and (iii) having signed a written informed consent to participate.

#### Recruitment of the study population

Companies were recruited via a request by sector organizations, either by distributing e-mails, hard copy invitations, or by telephonic contact. Within the company, support is needed at the level of manager, supervisor and worker [31]. Therefore, at the start of the project, the senior management of the eight companies committed themselves to the project by signing a letter of intent. Additionally, they agreed that their workers (supervisors and blue collar workers) are allowed to participate in the program during working hours. The recruitment of workers was conducted by the principal researcher through the usual and available communication channels within the companies, e.g., intranet, a personal letter or verbally. Finally, the principal researcher (EvD) informed all workers at the worksites regarding study and distributed a letter with the content of the study.

#### Randomization and blinding

In order to avoid intervention group contamination, to obtain maximal co-operation of managers and construction workers, and to enhance participants' compliance, cluster randomization at the level of the company is considered the best randomization strategy for this study. Randomization is performed by the principal researcher (EvD) using an electronic randomization tool ( http://www.randomizer.org) after the baseline measurement. Because the intervention takes place at the worksites, participating workers, managers, supervisors, as well as researchers/trainers cannot be blinded to the group assignment.

#### Sample size

The sample size was calculated as the number of workers needed to identify an effect of the intervention on occupational exposure to quartz. A previous intervention study on occupational exposure to wood dust aimed to decrease the exposure by 30% [34]. Assuming alpha is 0.05 and a power of 80% with a possibility to detect at least a 30% change in exposure in our intervention group, a sample size of 42 workers is needed. Although no specific information about exposure trends exists, it is well known that most occupational exposures show long-term downward trends of a few percent annually over recent decades [35]. Assuming this is also the case for the Dutch construction industry, a decline in exposure in the control group over the course of this study was taken into account. Based on these calculations, and taking into account a loss-to-follow-up of 20%, it was estimated that 60 construction workers are necessary in both the intervention and control group.

#### Co-interventions

Researchers emphasized to the managers that participation in other intervention studies or programs aimed at reducing occupational quartz exposure might influence the study results. Managers were therefore asked to not actively initiate other activities to decrease occupational exposure to quartz during the intervention period. After the intervention, managers are asked if any other intervention took place during the period of the current study.

#### Primary outcome measure

Respirable dust and quartz exposure are measured by collecting full-shift personal air samples from construction workers at different worksites [22]. These samples are collected using Dewell-Higgins cyclones mounted with a PVC filter (Milipore, pore size 5.0 μm, diameter 25 mm), connected to a calibrated Gillian GilAir pump with an airflow of 2 l/min. Cyclones are attached in the breathing zone of the worker. The amount of dust on filters is determined gravimetrically by pre- and post-weighing of filters on an analytical balance in a conditioned room [36]. Quartz is determined by infrared spectroscopy and X-ray diffraction [37]. Throughout their shift, workers are observed using a structured walk-through survey to obtain detailed information on work activities (including task time), workspace, work practices, the type of tools used, type of material worked on, and respiratory protective equipment [22].

#### Secondary outcomes

##### Behavioural determinants

Behavioural determinants related to respirable dust and quartz exposure are assessed by means of a questionnaire [22].

Questions about beliefs, motivation, risk perception and social influence are formulated based on a structure of questions often applied in the assessment of behavioral determinants [38]. Belief is defined as the indication of an individual’s belief in the effectiveness of various exposure controls and dust-reducing work practices, and is assessed by ten questions with answers on a 5-point scale. Additionally, motivation is measured by eleven questions with answers on a 5-point scale. Motivation is described as the indication of an individual’s intention (or motivation) to use exposure controls and dust-reducing work practices. Risk perception (six questions with answers on a 5-points scale) is defined as an indication of an individual’s perception of risk of quartz exposure and susceptibility to short- or long-term health effects. Lastly, three questions on a 5-point answer scale are constructed for social influence (i.e., indication whether an individual is influenced by co-workers or supervising personnel to use exposure controls and dust-reducing work practices).

An adapted format of an existing standardized scale is applied for risk propensity, which is an estimation of an individual’s inclination to seek or avoid general health risks [39]. This scale consists of six questions with a range from 1 to 5.

Knowledge is described as an estimation of an individual’s knowledge regarding exposure sources, technical control measures, substances, routes of exposure and short- and long-term effect of quartz dust and is assessed by eight quartz dust-related questions which are specifically composed for the current study, with answers on a dichotomous scale.

##### Organizational factors

Managers of all participating companies receive a questionnaire in order to gain insight into the occupational safety and health policy [22]. This questionnaire is based on the Occupational Health and Safety Assessment Series (OHSAS) norm for health and safety management, and specifically focuses on hazardous substances [23]. The following themes were included: (i) the presence and compliance of work procedures and workplace instructions; (ii) the extent to which training and education is offered to employees; (iii) the presence of management support towards a proactive health and safety culture (e.g.., toolbox meetings on a fixed and regular schedule to stimulate discussion between managers and construction workers); and (iv) the method of communicating and providing feedback with equipment contractors to improve services.

##### Technical control measures

Observations of the workers are used to assess to what extent technical control measures are used. Construction workers from the intervention and control group are observed throughout their shift on their use of technical control measures and protective equipment.

#### Other variables

Socio-demographic data such as gender, age, education level, job category, job history, and job experience are assessed at baseline measurement.

#### Evaluation

The ‘Relieved Working’ study will be evaluated by an effect evaluation and a process evaluation.

##### Effect evaluation

The potential difference between the baseline and follow-up values in the primary and secondary outcomes will be compared between the intervention group and the control group. Analyses of measurements at individual level (i.e., exposure assessment and questionnaire) will be performed by means of multi-level analyses taking clustering of observations of workers within the same company into account, as well as repeated measurements within one worker [40]. Due to randomization at company level, the data will be analyzed at three levels: time, worker and company. The multi-level analyses using the follow-up measurement as dependent variable will be adjusted for possible confounding factors, such as education level and job category. These variables will also be checked for effect modification.

##### Process evaluation

A process evaluation will be carried out to gain insight into the factors influencing the effectiveness of the intervention program of the ‘Relieved Working’ study. The process evaluation will assess seven aspects of the intervention, following the guidelines of Steckler and Linnan (2002): recruitment (sources and procedures used to recruit companies and construction workers), reach (attendance rates of construction workers), dose delivered (the amount of intervention components actually delivered), dose received (the extent to which construction workers use materials or components), fidelity (the extent to which the program was delivered as planned), satisfaction (extent to which the workers were satisfied with the overall content and the specific program components), and context (organizational and environmental characteristics that affect the intervention) [41, 42]. Reach and recruitment will be evaluated by data collected in logs from the commencement of the project. Two of the aspects, dose delivered and dose received, will be assessed by checklists completed by the principal researcher during the separate sessions in each company. Fidelity and satisfaction will be obtained by logs from the principal researcher and questionnaires at the end of the intervention for managers and construction workers. Context factors will be discussed in the project team and with the managers.

## Discussion

In this paper, the development of the ‘Relieved Working’ study and the design of its evaluation are described. This multidimensional intervention aims to establish an increase in the (proper) use of technical control measures by targeting both the workers’ behavior and organizational factors in order to significantly reduce occupational respirable dust and quartz exposure in the construction industry.

To our knowledge, this is the first study that will evaluate a multidimensional intervention in the construction industry tailored to both construction workers and managers. Since developing complex interventions, as in the present study, is a considerable challenge [43], intervention mapping was used to design an intervention which is not only tailored to the needs of the target population, but also to the abilities and opportunities of managers and implementers. A strength in applying intervention mapping in this study was that quantitative (i.e., baseline study) and qualitative information of construction workers was systematically collected and combined with scientific literature to tailor the intervention to their needs. By also involving managers in the development, it eventuated that managers played an important role in the availability of technical control measures at all worksites. An additional informative session is therefore organized for all managers. Involving all stakeholders in the development of the intervention was another strength, as it provided insight into the practical strategies. For example, the intervention is incorporated within the existing ‘Toolbox Education System’ of the construction industry. Because this education system is obligatory, a high level of commitment is expected from senior management, supervisors and construction workers. Another strength of the study is the extensive evaluation in terms of both effect and process evaluation. The effect evaluation will include personal exposure assessment, as well as behavioral and organizational outcomes, which could provide meaningful results at worker, company and policy level (e.g., guidelines). A thorough process evaluation alongside a cluster RCT will provide more detailed information regarding the context and degree of the implementation of the intervention [44]. A process evaluation may help researchers distinguishing between interventions that are not effective due to their intervention protocol and underlying theories, and those that are not implemented adequately [44, 45].

Some limitations also need to be considered with respect to the development and design of the study. Firstly, as already mentioned in previous intervention studies in the construction industry [46, 47], intervention mapping is a time-consuming process. To be efficient, qualitative data (interviews and focus groups) were combined with quantitative data (observation forms and questionnaires among construction workers and managers) in the current study. However, the development of the intervention was still time-consuming and step 3 of intervention mapping was not fully applied. Secondly, the construction companies who commit themselves to the project are probably early adopters when it comes to health and safety [48]. The participating construction workers, and especially the managers, have more sympathy towards the program than is general shown throughout in the construction industry. Employers of these companies might therefore be more aware of the hazards in the construction industry. The third limitation is that the cluster RCT is two-armed (control versus intervention), whereas the intervention consists of several components. This design does not allow separate evaluation of each intervention component in terms of effectiveness. The process evaluation will focus on the different components and their working mechanism of the components in view of the entire program.

In conclusion, the ‘Relieved Working’ study was developed to establish an increase in the proper use of technical control measures in the construction industry by targeting both the workers’ behaviour and organizational factors. Applying Intervention Mapping in the development of an intervention to reduce occupational quartz exposure was useful, as different stakeholders provide input for the intervention as well as the implementation strategy. Therefore, it is likely that this program fits the target population well, and increases the likelihood of compliance and effectiveness. Alongside the development of the intervention, this paper also described the design of a cluster RCT to determine the effectiveness of this program. This insight can play an important role in the decisions of employers in regard to investing in such interventions in the future.
